# Let-7c-3p Suppresses Ovarian Cancer Progression by Targeting S100A6 and Inhibiting JAK-STAT Signaling

**DOI:** 10.3390/cancers18172834

**Published:** 2026-09-01

**Authors:** Bing Yuan, Xiaoyan Huang, Juan Li, Yujie Su, Shan Li, Hang Yuan, Yuli Bi, Panpan Bei, Jidong Wang, Mingyong Han

**Affiliations:** 1 Cancer Therapy and Research Center, Shandong Provincial Hospital, Shandong University, Jinan 250021, China; yuanbing@qdu.edu.cn; 2 Department of Gynecology, Shandong Provincial Maternal and Child Health Care Hospital, Qingdao University, Jinan 250014, China; huangxiaoyan@sdsfyb.cn (X.H.); noname7@sdsfyb.cn (J.L.); suyujie@sdsfyb.cn (Y.S.); lishan2@qdu.edu.cn (S.L.); yuanhang1@qdu.edu.cn (H.Y.); biyuli@sdsfyb.cn (Y.B.); beipanpan@sdsfyb.cn (P.B.)

**Keywords:** ovarian cancer, S100A6, Let-7c-3p, JAK-STAT pathway, miRNA, proliferation, migration, invasion

## Abstract

**Background/Objectives**: Ovarian cancer is one of the most lethal gynecological malignancies worldwide, largely due to late diagnosis and rapid disease progression. Increasing evidence suggests that dysregulation of calcium-binding proteins and microRNA-mediated regulatory networks contributes to ovarian cancer progression. However, the role of S100A6 and its upstream regulatory mechanisms in ovarian cancer remain poorly understood. **Methods**: S100A6 expression in ovarian cancer cell lines and tissues was assessed by transcriptome sequencing, qPCR, and immunohistochemistry. Functional assays (CCK-8, EdU, Transwell, wound-healing, flow cytometry) and a xenograft model were used to evaluate S100A6 effects. Bioinformatic analysis, dual-luciferase reporter assays, and rescue experiments identified upstream miRNAs. JAK-STAT involvement was examined by Western blot. Survival analysis was performed using Kaplan–Meier Plotter. **Results**: S100A6 expression was significantly upregulated in ovarian cancer cells and tumor tissues compared with normal controls. Silencing S100A6 inhibited cell proliferation, migration, and invasion while promoting apoptosis in vitro and suppressed tumor growth in vivo. Mechanistically, S100A6 activated the JAK–STAT pathway, and pharmacological inhibition of STAT3 with Stattic reversed S100A6-promoted migration. Further analyses identified Let-7c-3p as a direct upstream regulator of S100A6. Let-7c-3p bound to the 3′-UTR of S100A6 and suppressed its expression, thereby reversing S100A6-mediated oncogenic phenotypes and inhibiting JAK-STAT signaling. Survival analysis revealed that elevated S100A6 expression was associated with shorter progression-free survival in ovarian cancer patients. **Conclusions**: Our findings identify a novel Let-7c-3p/S100A6/JAK-STAT regulatory axis that promotes ovarian cancer progression. This study provides new insights into the molecular mechanisms underlying ovarian cancer development and suggests that targeting this regulatory network may represent a potential therapeutic strategy.

## Simple Summary

Ovarian cancer is a highly aggressive disease often diagnosed at advanced stages, leading to limited treatment options and poor patient outcomes. Identifying key molecules that drive tumor growth and spread may help develop more effective diagnostic and therapeutic strategies. In this study, we investigated the role of a protein called S100A6 in ovarian cancer cells and tumor tissues. Our findings show that S100A6 is present at elevated levels in ovarian cancer and promotes cancer cell proliferation, migration, and invasion while suppressing cell death. We further identified a small regulatory RNA molecule, Let-7c-3p, that directly suppresses S100A6 production, and we uncovered that S100A6 exerts its effects by activating a cellular communication pathway known as JAK-STAT. Importantly, restoring Let-7c-3p function reversed the cancer-promoting effects of S100A6. These results highlight a new regulatory axis that drives ovarian cancer progression and suggest that targeting this pathway may offer new opportunities for diagnosis and treatment of this devastating disease.

## 1. Introduction

Ovarian cancer remains one of the most lethal gynecological malignancies worldwide and is a leading cause of cancer-related death among women. The high mortality rate is largely attributable to late-stage diagnosis, rapid disease progression, and the frequent development of chemoresistance [1,2]. Despite advances in cytoreductive surgery and platinum-based chemotherapy, the overall prognosis of patients with advanced ovarian cancer remains poor, with five-year survival rates below 30% [3,4]. Therefore, identifying novel molecular mechanisms underlying ovarian cancer progression is essential for improving early diagnosis and developing more effective therapeutic strategies [5].

Accumulating evidence suggests that dysregulation of calcium-binding proteins and oncogenic signaling pathways plays an important role in ovarian cancer development. The S100 protein family consists of more than 20 small calcium-binding proteins that regulate diverse cellular processes, including cell proliferation, migration, apoptosis, and cytoskeletal organization [6]. Aberrant expression of several S100 family members has been reported in multiple malignancies, including ovarian cancer [7]. For example, S100A4 has been implicated in tumor metastasis [8], while S100A1 overexpression has been associated with enhanced tumor growth and poor clinical outcomes [9]. Among these proteins, S100A6 has attracted increasing attention because of its involvement in tumorigenesis and metastasis in several cancer types [10]. Early work by Wei et al. demonstrated that serum S100A6 concentration correlates with peritoneal tumor burden in mouse models and is elevated in patients with advanced-stage disease, suggesting its potential utility as a biomarker for disease monitoring [11]. However, a more recent comprehensive analysis of S100 family members in a large cohort of 462 ovarian tumors revealed a nuanced picture: high cytoplasmic and nuclear S100A6 protein expression was paradoxically associated with better overall survival, whereas mRNA levels showed no prognostic significance [12]. These contrasting findings underscore the need for further mechanistic studies to delineate the context-dependent and possibly post-transcriptionally regulated functions of S100A6 in ovarian cancer progression.

MicroRNAs (miRNAs) are a class of small noncoding RNAs that regulate gene expression at the post-transcriptional level and play critical roles in tumor initiation and progression [13]. Numerous studies have shown that dysregulation of miRNAs contributes to ovarian cancer development through the modulation of oncogenic signaling pathways, epithelial–mesenchymal transition, and therapeutic resistance [14,15]. The let-7 family is one of the best-characterized tumor-suppressive miRNA families and has been reported to inhibit tumor growth and metastasis in various cancers. Reduced expression of let-7 members has been associated with increased tumor aggressiveness and poor prognosis [16,17]. However, whether Let-7c-3p regulates S100A6 expression and contributes to ovarian cancer progression has not been investigated.

In the present study, we explored the biological role of S100A6 in ovarian cancer and investigated its upstream regulatory mechanism. We demonstrate that S100A6 is significantly upregulated in ovarian cancer cells and tissues and promotes tumor cell proliferation, migration, and invasion. Mechanistically, we identify Let-7c-3p as a direct upstream regulator of S100A6 and show that the Let-7c-3p/S100A6 axis modulates ovarian cancer progression through activation of the JAK-STAT signaling pathway. These findings reveal a previously unrecognized regulatory network in ovarian cancer and provide new insights into the molecular mechanisms driving disease progression.

## 2. Materials and Methods

### 2.1. Cell Lines and Culture

The cell lines SKOV3, A2780, and IOSE80 were obtained from commercial sources. SKOV3 was purchased from IMMOCELL (Xiamen, China), with the original source being the American Type Culture Collection (ATCC; catalog No. HTB-77). A2780 and IOSE80 were bought from FUHENG BIOLOGY (Shanghai, China). A2780 originates from the Expert Protein Analysis System (ExPASy; catalog No. CVCL_0134), while IOSE80 originates from the Leibniz Institute DSMZ-German Collection of Microorganisms and Cell Cultures (DSMZ; no catalog number supplied). A2780 and SKOV3 cells were cultured in RPMI-1640 medium (Thermo Fisher Scientific, Waltham, MA, USA) supplemented with 10% fetal bovine serum (FBS; Thermo Fisher Scientific, Waltham, MA, USA), 100 U/mL penicillin, and 100 μg/mL streptomycin. IOSE80 cells were maintained in DMEM medium with 10% FBS and growth supplements as recommended. All cells were incubated at 37 °C in a humidified atmosphere containing 5% CO_2_.

### 2.2. Transcriptome Sequencing and Analysis

Total RNA was extracted from IOSE80 and SKOV3 cells using TRIzol reagent (Invitrogen, Carlsbad, CA, USA)according to the manufacturer’s protocol. RNA integrity was assessed using an Agilent 2100 Bioanalyzer (Agilent Technologies, Santa Clara, CA, USA). Libraries were prepared using the Illumina TruSeq RNA Sample Prep Kit (Illumina, San Diego, CA, USA) and sequenced on an Illumina HiSeq platform (2 × 150 bp paired-end reads). Raw reads were filtered for quality and aligned to the human reference genome (GRCh38) using HISAT2. Raw read counts were generated for each gene and used for differential expression analysis with DESeq2. Gene expression levels were normalized and visualized as FPKM values.

### 2.3. Quantitative Real-Time PCR (qRT-PCR)

Total RNA was reverse-transcribed using the PrimeScript RT Reagent Kit (Cwbio, Beijing, China). qPCR was performed using SYBR Premix Ex Taq (Cwbio) on a LightCycler 480 System (Hehuibiotech, Suzhou, China). The primers for S100A6 were forward 5′-AAGCACACCCTGAGCAAGAA-3′ and reverse 5′-CACCTCCTGGTCCTTGTTCC-3′. GAPDH was used as an internal control. Relative expression levels were calculated using the 2^−ΔΔCt^ method. Each sample was analyzed in triplicate.

### 2.4. Immunohistochemistry (IHC) on Tissue Microarray

Ovarian cancer tissue microarrays (TMAs) containing 88 ovarian cancer tissues and 8 normal ovarian tissues were obtained from Novus Biologicals. Sections (4 μm) were deparaffinized, rehydrated, and subjected to antigen retrieval in citrate buffer (pH 6.0). Endogenous peroxidase was blocked with 3% H_2_O_2_. After blocking with 5% goat serum, slides were incubated with anti-S100A6 antibody (1:200, Abclonal, Wuhan, China) overnight at 4 °C. Detection was performed using HRP-conjugated secondary antibody and DAB substrate. Staining intensity was scored semi-quantitatively by two independent pathologists based on the proportion of positive cells and staining intensity.

### 2.5. siRNA Transfection

Small interfering RNAs (siRNAs) targeting S100A6 si-S100A6-1, si-S100A6-3 and negative control siRNA (si-NC) were synthesized by GENEWIZ. A2780 and SKOV3 cells were seeded in 6-well plates and transfected with 50 nM siRNA using Lipofectamine RNAiMAX (Invitrogen) according to the manufacturer’s instructions. Knockdown efficiency was confirmed by qRT-PCR and Western blot 48 h post-transfection.

### 2.6. Cell Proliferation Assays

CCK-8 assay: Cells were seeded in 96-well plates at 2 × 10^3^ cells/well. At indicated time points (0, 24, 48, 72 h), 10 μL of CCK-8 solution (MedChemExpress, Monmouth Junction, NJ, USA) was added to each well and incubated for 2 h. Absorbance at 450 nm was measured using a microplate reader. EdU assay: Cell proliferation was assessed using the Click-iT EdU Imaging Kit (Invitrogen). EdU (10 μM) was added to the culture medium for 2 h. Cells were fixed, permeabilized, and stained according to the manufacturer’s protocol. Nuclei were counterstained with Hoechst 33342. EdU-positive cells were counted under a fluorescence microscope.

### 2.7. Cell Migration and Invasion Assays

Transwell migration assay: Transwell chambers (8 μm pore size, Corning, Corning, NY, USA) were placed in 24-well plates. Cells (5 × 10^4^ in serum-free medium) were seeded in the upper chamber, and medium containing 10% FBS was added to the lower chamber. After 24 h incubation, cells on the upper surface were removed, and migrated cells on the lower surface were fixed, stained with 0.1% crystal violet, and counted in five random fields. Transwell invasion assay: The upper chamber was pre-coated with Matrigel (BD Biosciences) diluted in serum-free medium. Cells (1 × 10^5^) were seeded, and the procedure was similar to the migration assay. Wound healing assay: Cells were grown to confluence in 6-well plates, and a scratch was made with a 200 μL pipette tip. Debris was removed by washing with PBS. Images were captured at 0 and 24 h, and wound closure was analyzed using ImageJ software (1.54r).

### 2.8. Apoptosis Assay

Apoptosis was evaluated using the Annexin V-FITC/PI Apoptosis Detection Kit (BD Biosciences). Cells were harvested 48 h after transfection, washed with cold PBS, and resuspended in binding buffer. Annexin V-FITC and PI were added and incubated for 15 min in the dark. Stained cells were analyzed by flow cytometry (BD FACSCalibur, (BD Biosciences, Franklin Lakes, NJ, USA) within 1 h. Early and late apoptotic cells were quantified.

### 2.9. In Vivo Tumorigenicity Assay

All animal experiments were approved by the Ethics Committee of Shandong Provincial Maternal and Child Health Care Hospital. Female BALB/c nude mice (4–6 weeks old) were randomly divided into three groups (*n* = 5 per group). A2780 cells stably transfected with shRNA1, shRNA3 or sh-NC (5 × 10^6^ cells in 0.1 mL PBS) were subcutaneously injected into the right flank of each mouse. Tumor volume was measured every 3 days using calipers and calculated as (length × width^2^)/2. After 26 days, mice were sacrificed, and tumors were excised and weighed.

### 2.10. Western Blot Analysis

Cells were lysed in RIPA buffer containing protease and phosphatase inhibitors. Protein concentration was determined by BCA assay. Equal amounts of protein (30 μg) were separated by SDS-PAGE and transferred to PVDF membranes. Membranes were blocked with 5% non-fat milk and incubated overnight at 4 °C with primary antibodies against S100A6, JAK2, p-JAK2, STAT3, p-STAT3, and GAPDH (all antibodies from Cell Signaling Technology, Danvers, MA, USA). After washing, membranes were incubated with HRP-conjugated secondary antibodies. Bands were visualized using ECL reagent and quantified by densitometry using ImageJ.

### 2.11. Plasmid Transfection and Stattic Treatment

For transient overexpression, SKOV3 and A2780 cells were seeded in 6-well plates at 60–70% confluence and transfected with pcDNA-S100A6 or pcDNA-NC using Lipofectamine 3000 (Invitrogen) according to the manufacturer’s instructions. At 24 h post-transfection, cells were treated with Stattic (Cat. No. HY-13818; MedChemExpress, Monmouth Junction, NJ, USA) at 10 μM for 24 h, while control cells received an equal volume of DMSO.

### 2.12. miRNA Target Prediction and Luciferase Reporter Assay

Potential upstream miRNAs targeting S100A6 were predicted using TargetScan, miRDB, and miRTarBase. Five candidate miRNAs were selected for preliminary screening. Among them, miR-let-7c-3p was prioritized for luciferase validation because the Let-7 family has been extensively implicated as tumor suppressors in various cancers, and interactions between Let-7 members and S100 proteins have been previously suggested [18]. The 3′-UTR of S100A6 containing the putative binding site for Let-7c-3p was cloned into the pmirGLO dual-luciferase reporter vector (Promega, Madison, WI, USA). A mutant reporter was generated by site-directed mutagenesis. HEK293T cells were co-transfected with a wild-type or mutant reporter and Let-7c-3p mimics or a negative control using Lipofectamine 2000. After 48 h, firefly and Renilla luciferase activities were measured using the Dual-Luciferase Reporter Assay System (Promega). Renilla luciferase activity was normalized to firefly luciferase activity.

### 2.13. miRNA Mimic Transfection and Rescue Experiments

miRNA Let-7c-3p mimics and the negative control (miR-NC) were synthesized by GENEWIZ. For rescue experiments, SKOV3 cells were divided into four groups: (1) pcDNA-NC + miR-NC, (2) pcDNA-S100A6 + miR-NC, (3) pcDNA-NC + miR-let-7c, (4) pcDNA-S100A6 + miR-let-7c. pcDNA-S100A6 overexpressing plasmid was constructed by cloning the full-length S100A6 coding sequence into pcDNA3.1. Transfections were performed using Lipofectamine 3000. After 48 h, cells were harvested for functional assays and Western blot analysis as described above.

### 2.14. Kaplan–Meier Survival Analysis

Survival analysis was performed using the Kaplan–Meier Plotter database (http://kmplot.com), an online platform that integrates gene expression and survival data from The Cancer Genome Atlas (TCGA), Gene Expression Omnibus (GEO), and European Genome-phenome Archive (EGA). The ovarian cancer cohort mainly consists of high-grade serous ovarian cancer cases with available survival information.

S100A6 expression was analyzed using the Affymetrix probe ID 217728_at. Progression-free survival (PFS) was selected as the primary endpoint. Patients were stratified into high- and low-expression groups according to the auto-selected best cutoff determined by the platform. Hazard ratios (HRs) with 95% confidence intervals (CIs) and log-rank *p* values were automatically calculated using the Cox proportional hazards model. A *p* value < 0.05 was considered statistically significant.

### 2.15. Statistical Analysis

All data are presented as mean ± standard deviation (SD) from at least three independent experiments. Statistical analyses were performed using GraphPad Prism 9.0. Comparisons between two groups were made using Student’s t-test, and among multiple groups using one-way ANOVA followed by Tukey’s post hoc test. The assumption of normal distribution and homogeneity of variance were tested prior to ANOVA. *p* < 0.05 was considered statistically significant. *, **, and *** indicate *p* < 0.05, *p* < 0.01, and *p* < 0.001, respectively.

### 2.16. Ethical Considerations

This research was carried out in compliance with the principles of the Declaration of Helsinki. The Ethics Committee of Shandong Provincial Maternal and Child Health Care Hospital granted its approval (Reference No. 2023-042) on 20 November 2023.

## 3. Results

### 3.1. Identification of Candidate Genes in Ovarian Cancer Cells

**Figure 1:**
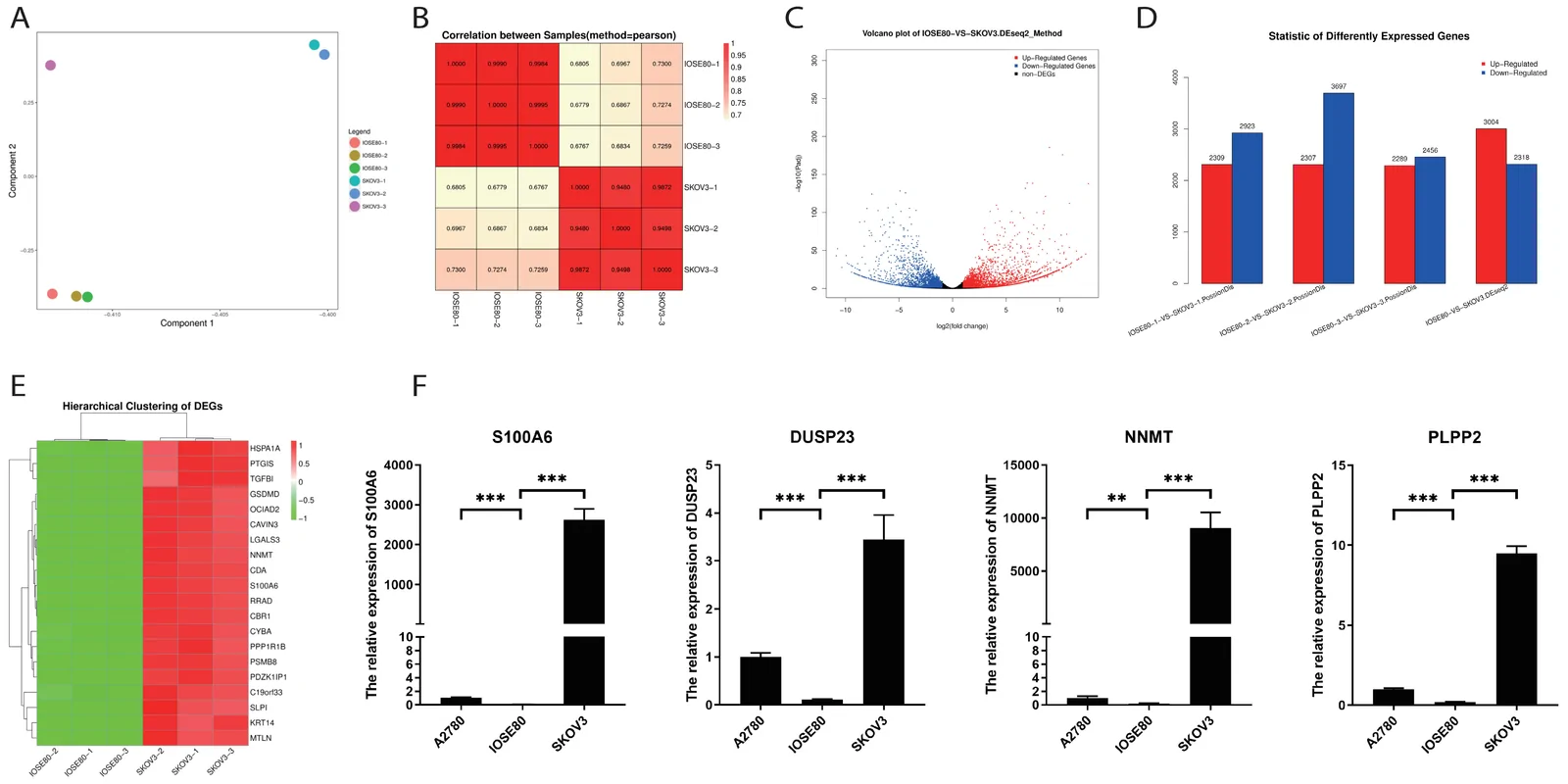
Identification and validation of upregulated genes in ovarian cancer cells. (**A**) Principal component analysis (PCA) plot showing the overall distribution of transcriptomic profiles among IOSE80 (normal ovarian epithelial) and SKOV3 (ovarian cancer) cells. (**B**) Sample correlation heatmap indicating the similarity of biological replicates within and between cell lines. (**C**) Volcano plot of differentially expressed genes between ovarian cancer cells and normal controls. Upregulated genes are shown in red, downregulated genes in blue, and genes with no significant change in black (|log_2_FC| ≥ 1, adjusted *p* < 0.05). (**D**) Bar chart summarizing the numbers of significantly upregulated and downregulated genes in SKOV3 cells compared with IOSE80. (**E**) Heatmap of differentially expressed genes across the two cell lines. (**F**) Quantitative PCR validation of four selected candidate genes (S100A6, DUSP23, NNMT, PLPP2) in IOSE80, SKOV3, and A2780 cells. Expression levels were normalized to IOSE80. Data are mean ± SD from three independent experiments. ** *p* < 0.01, *** *p* < 0.001.

**Figure 2:**
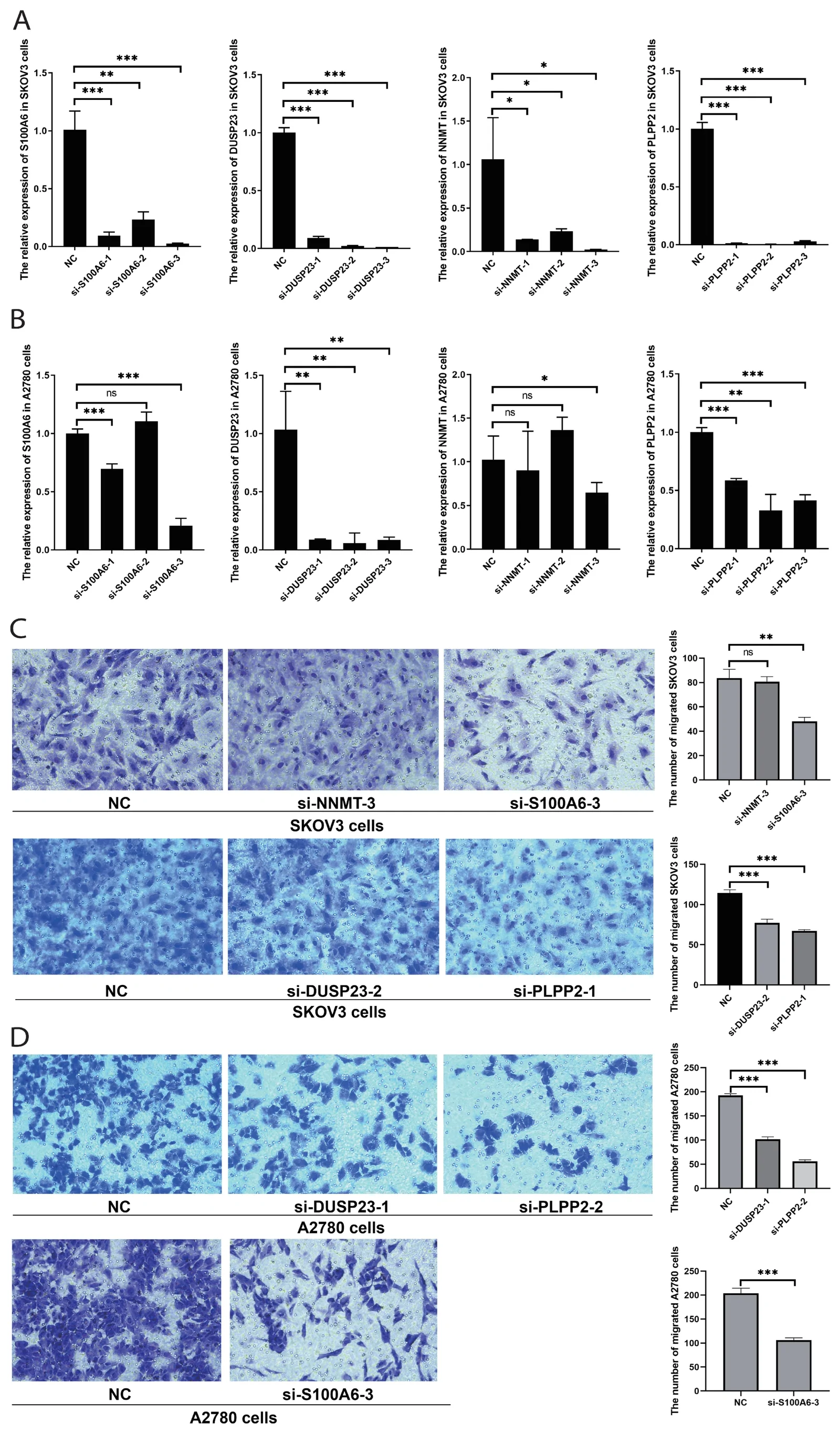
Functional screening of candidate genes by siRNA-mediated knockdown. (**A**,**B**) Knockdown efficiency of three independent siRNAs targeting S100A6, DUSP23, NNMT, and PLPP2 in SKOV3 (**A**) and A2780 (**B**) cells, as determined by quantitative PCR. Expression levels were normalized to the NC group. (**C**,**D**) Representative images and quantification of Transwell migration assays in SKOV3 (**C**) and A2780 (**D**) cells transfected with the selected siRNA for each target gene. * *p* < 0.05, ** *p* < 0.01, *** *p* < 0.001.

To identify key molecules involved in ovarian cancer progression, we performed transcriptome sequencing on the normal ovarian epithelial cell line IOSE80 and the ovarian cancer cell line SKOV3, followed by literature-based screening. Four candidate genes—S100A6, DUSP23, NNMT, and PLPP2—showed consistently elevated expression in cancer cells compared with normal ovarian epithelial cells (Figure 1A–E). Quantitative PCR confirmed that all four genes were upregulated in SKOV3 and A2780 cells relative to the IOSE80 control line (Figure 1F). We then designed three independent siRNAs for each gene and validated their knockdown efficiencies (Figure 2A,B). Functional screening via migration assays revealed that silencing of S100A6, DUSP23, or PLPP2 significantly reduced cell migration in both ovarian cancer cell lines (Figure 2C,D). Our literature search and preliminary functional data pointed to S100A6 as the most compelling candidate. We therefore pursued it for mechanistic studies.

### 3.2. S100A6 Upregulation Associates with Poorer Survival and Is Elevated in Ovarian Cancer Tissues

**Figure 3:**
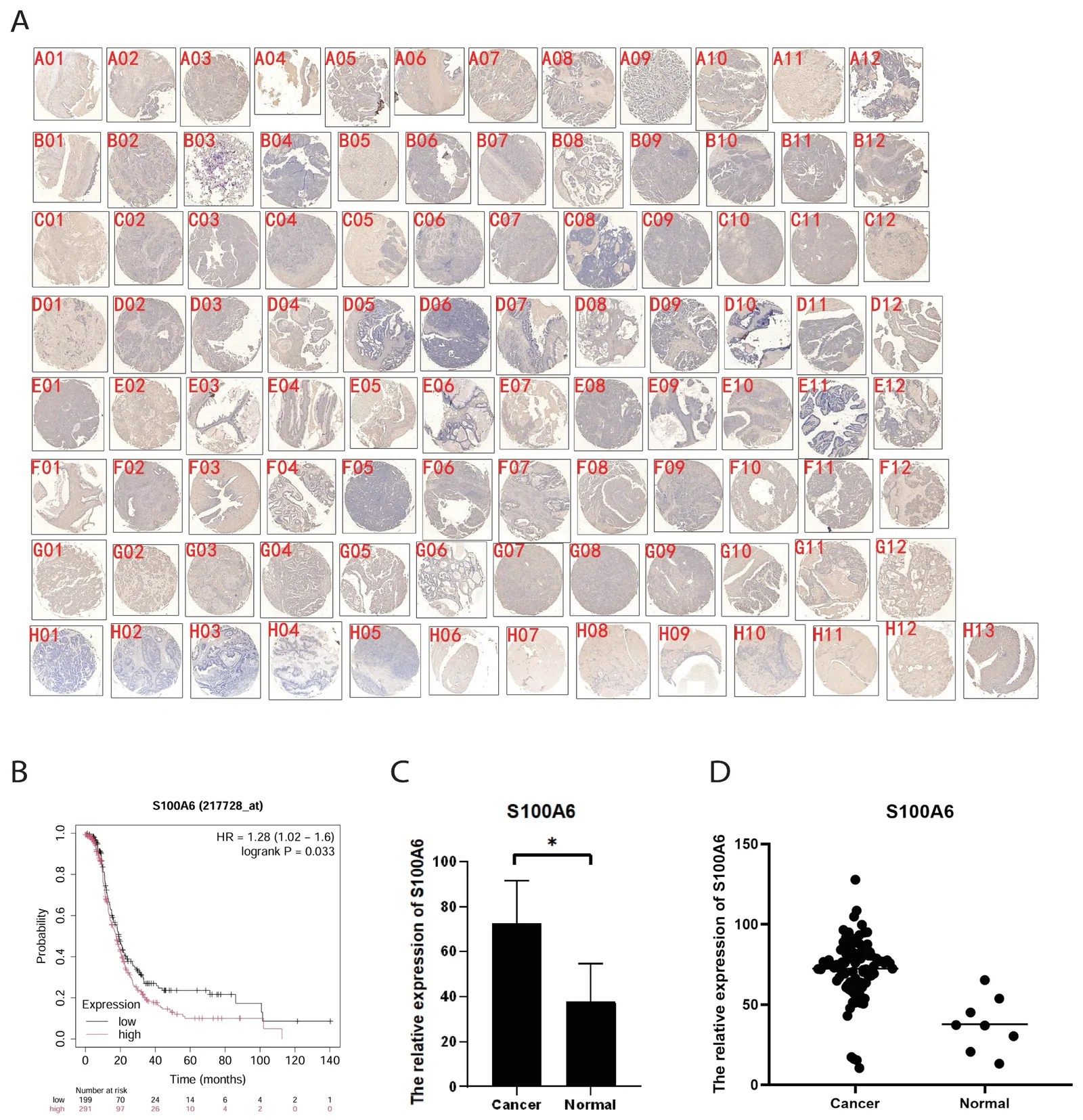
S100A6 expression correlates with poorer survival and is upregulated in ovarian cancer tissues. (**A**) Representative immunohistochemical images of S100A6 staining in normal ovarian tissues and ovarian cancer tissues. (**B**) Kaplan–Meier plot showing progression-free survival of ovarian cancer patients stratified by high versus low S100A6 expression (HR = 1.28, 95% CI: 1.02–1.60, *p* = 0.033). (**C**,**D**) Quantification of S100A6 immunohistochemical staining intensity in normal ovarian epithelium and ovarian cancer tissues, based on tissue microarrays containing 88 primary/metastatic ovarian cancer samples and 8 normal ovaries. * *p* < 0.05.

To evaluate the clinical relevance of S100A6 upregulation, we first interrogated the Kaplan–Meier Plotter database. Higher S100A6 mRNA levels were associated with shorter progression-free survival in ovarian cancer patients (HR = 1.28, 95% CI: 1.02–1.60, *p* = 0.033) (Figure 3A). We next examined S100A6 protein expression in tissue microarrays containing 88 primary and metastatic ovarian cancer samples and 8 normal ovarian tissues. Immunohistochemical staining revealed markedly stronger S100A6 staining in tumor tissues than in normal ovarian epithelium (Figure 3B–D). These clinical data demonstrate that S100A6 is upregulated at the protein level in ovarian cancer and that its elevated expression associates with shorter progression-free survival, reinforcing its potential role in disease progression.

### 3.3. S100A6 Promotes Malignant Phenotypes of Ovarian Cancer Cells

**Figure 4:**
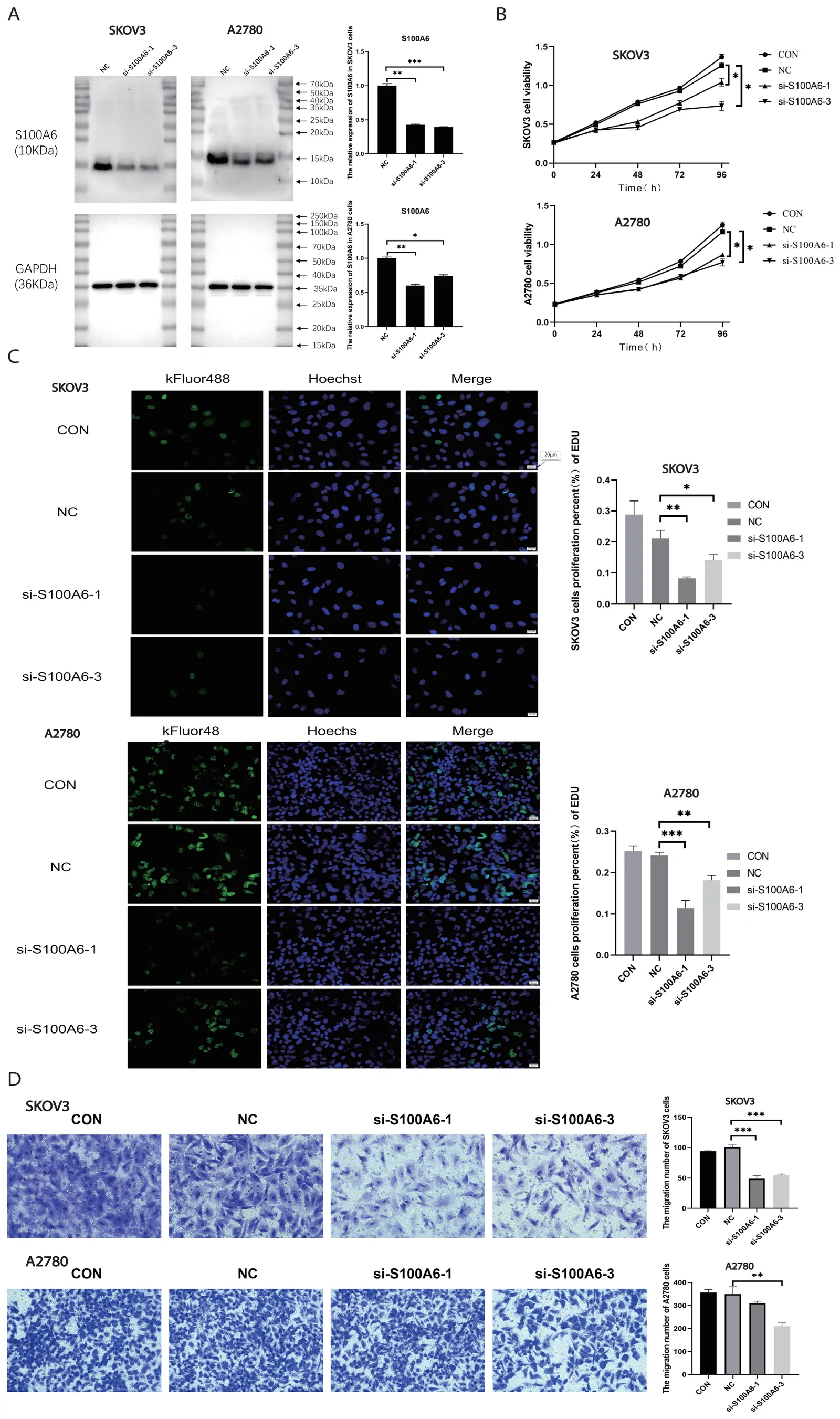

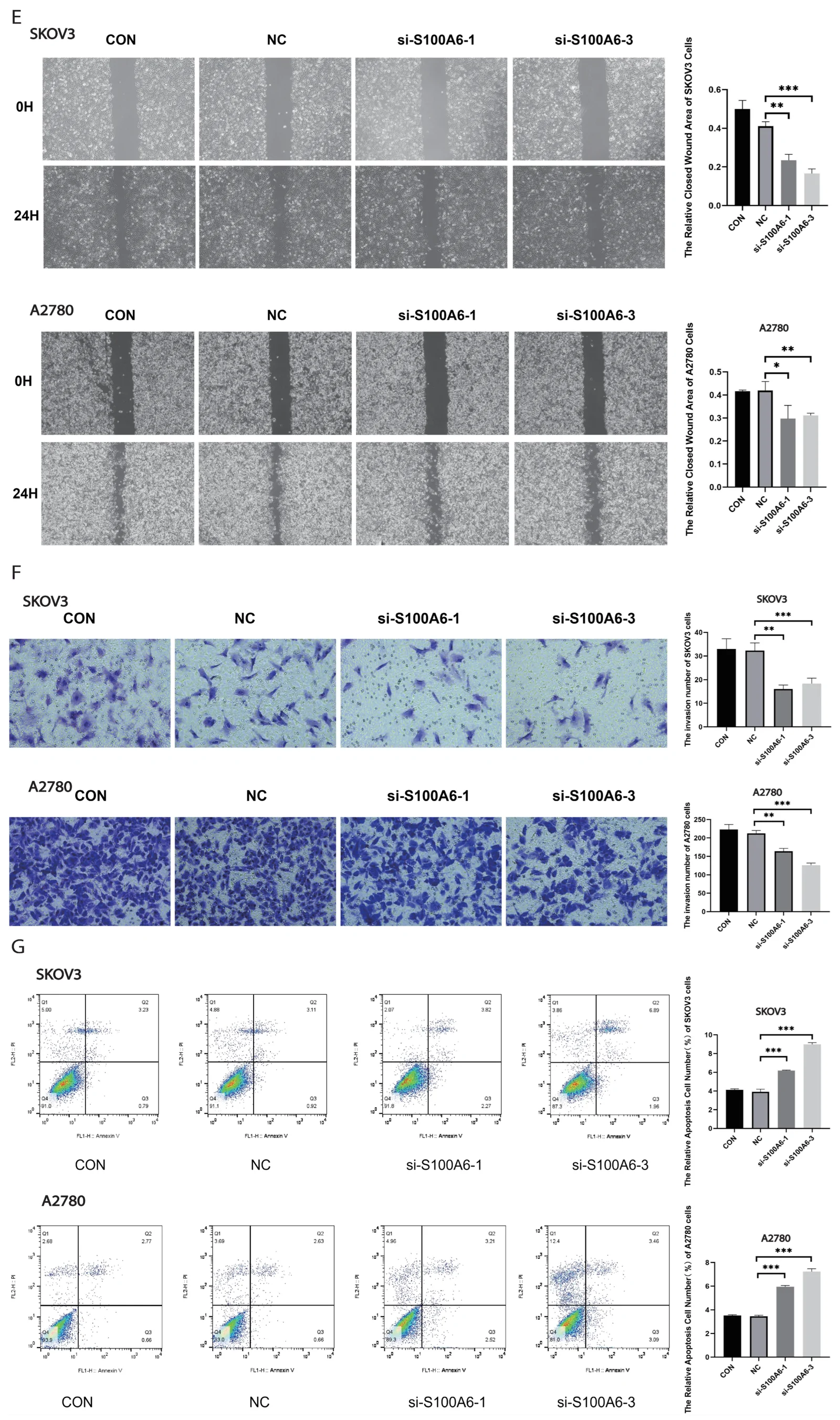
S100A6 regulates proliferation, migration, invasion, and apoptosis of ovarian cancer cells. (**A**) Western blot analysis of S100A6 protein levels in SKOV3 and A2780 cells transfected with si-NC or si-S100A6, with corresponding quantification. S100A6 expression was significantly reduced by siRNA knockdown in both cell lines. (**B**) CCK-8 assays showing reduced proliferation of SKOV3 and A2780 cells following S100A6 silencing. (**C**) EdU assays demonstrating decreased proliferation in SKOV3 and A2780 cells after S100A6 knockdown. (**D**) Transwell migration assays evaluating migration ability in SKOV3 and A2780 cells. (**E**) Wound-healing assays showing impaired migration capacity after S100A6 silencing in SKOV3 and A2780 cells. (**F**) Transwell invasion assays demonstrating reduced invasive ability in SKOV3 and A2780 cells following S100A6 knockdown. (**G**) Flow cytometry analysis showing increased apoptosis after S100A6 downregulation in SKOV3 and A2780 cells. Viable cells appear in the lower left quadrant. The lower right quadrant marks early apoptosis (annexin V^+^/PI^−^), the upper right quadrant late apoptosis (annexin V^+^/PI^+^), and the upper left quadrant necrosis (annexin V^−^/PI^+^). * *p* < 0.05, ** *p* < 0.01, *** *p* < 0.001.

**Figure 5:**
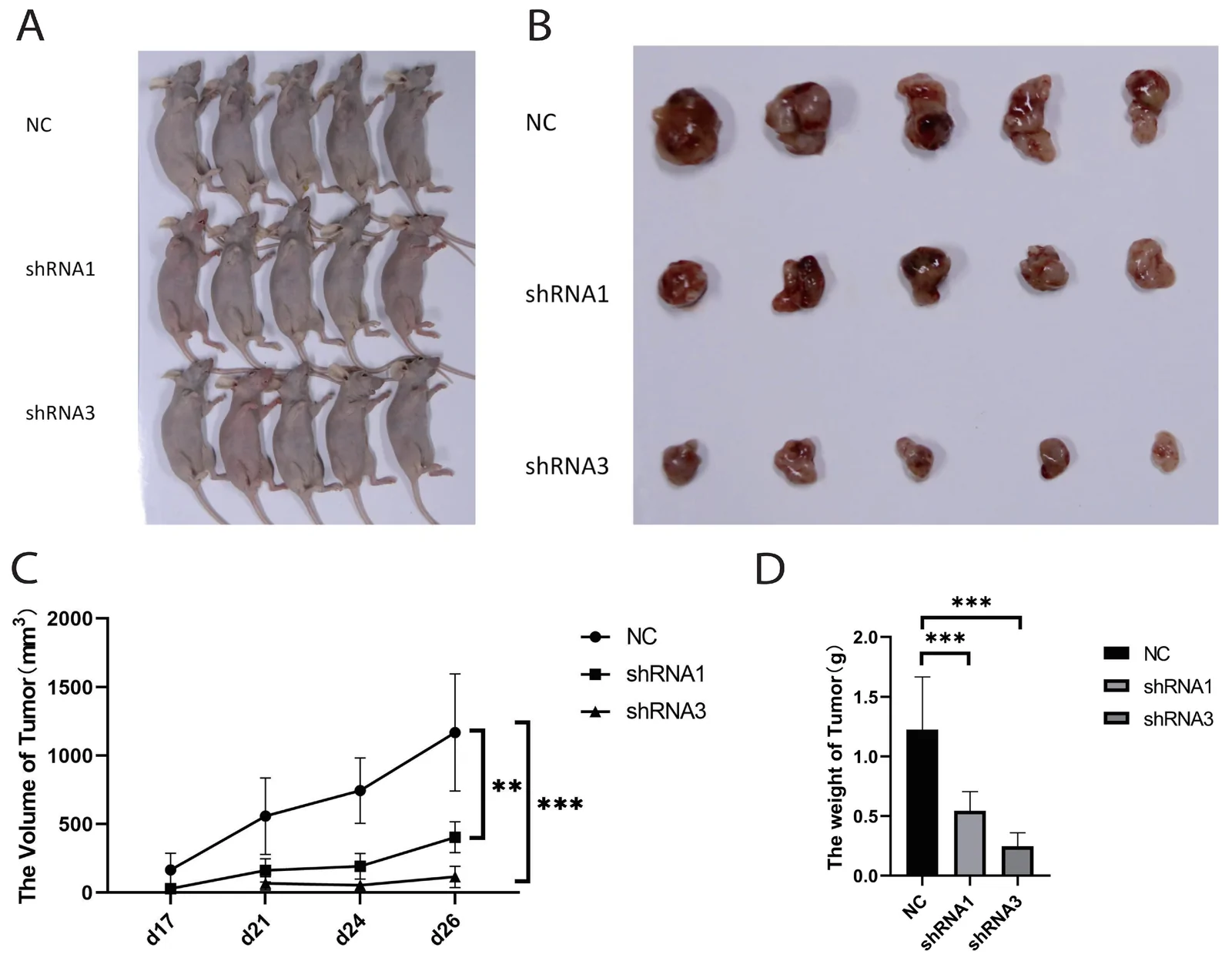
S100A6 knockdown suppresses tumor growth in a xenograft mouse model. (**A**) Images of mice before tumor implantation, showing the three experimental groups (NC, shRNA1, shRNA3; *n* = 5 per group). (**B**) Excised tumors harvested from each group at the end of the experiment. Tumors in the shRNA1 and shRNA3 groups were visibly smaller than those in the NC group. (**C**) Tumor volume curves measured at the indicated time points after implantation. Mice were sacrificed on day 21. Data are mean ± SD. *p* = 0.0047 (shRNA1 vs. NC), *p* = 0.00062 (shRNA3 vs. NC). (**D**) Tumor weights at harvest. The differences in tumor weight between the NC group and either shRNA1 or shRNA3 group were highly significant (*p* = 4.31 × 10^−11^ and *p* = 1.28 × 10^−11^, respectively), indicating that S100A6 knockdown effectively suppressed tumor growth in vivo. ** *p* < 0.01, *** *p* < 0.001.

Given the elevated expression of S100A6 in ovarian cancer, we next investigated its functional role in ovarian cancer cell behavior. We validated the knockdown efficiency of S100A6 siRNAs at the protein level by Western blot in both ovarian cancer cell lines, ensuring the reliability of subsequent functional assays (Figure 4A). Specific siRNAs targeting S100A6 were synthesized and transfected into ovarian cancer cells. CCK-8 and EdU assays revealed that knockdown of S100A6 significantly attenuated the proliferative capacity of ovarian cancer cells (Figure 4B,C). Transwell migration and wound healing assays demonstrated that S100A6 silencing markedly reduced cell migration (Figure 4D,E). Similarly, Matrigel invasion assays showed impaired invasive ability following S100A6 knockdown (Figure 4F). Flow cytometric analysis revealed a significant increase in apoptotic cell numbers upon S100A6 downregulation (Figure 4G). Furthermore, xenograft tumor models established by subcutaneous injection of S100A6-knockdown SKOV3 cells exhibited significantly reduced tumor volumes compared to control groups (Figure 5). Collectively, these results indicate that S100A6 promotes proliferation, migration, and invasion while suppressing apoptosis in ovarian cancer cells, and facilitates tumor growth in vivo.

### 3.4. S100A6 Activates the JAK-STAT Signaling Pathway

**Figure 6:**
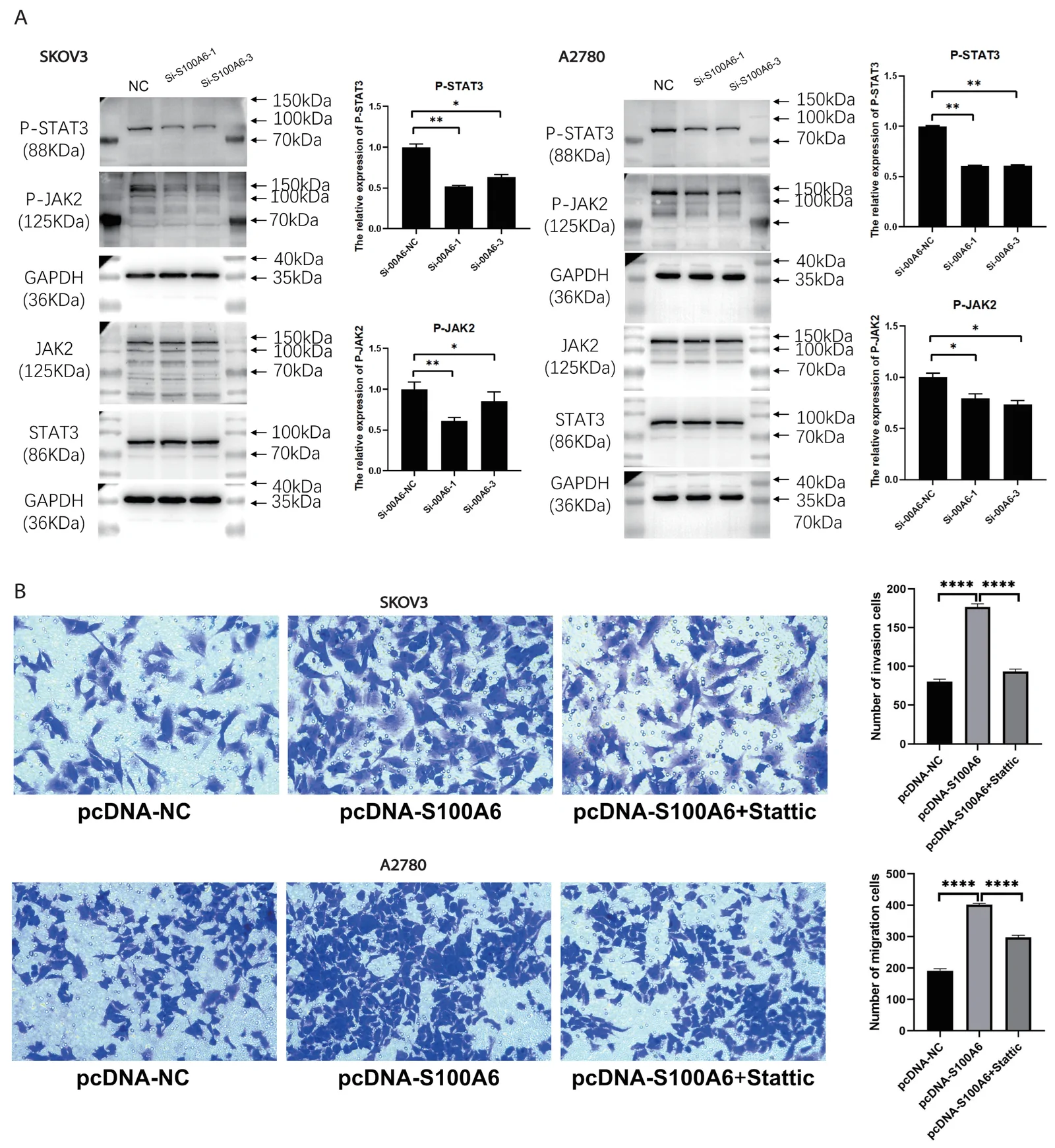

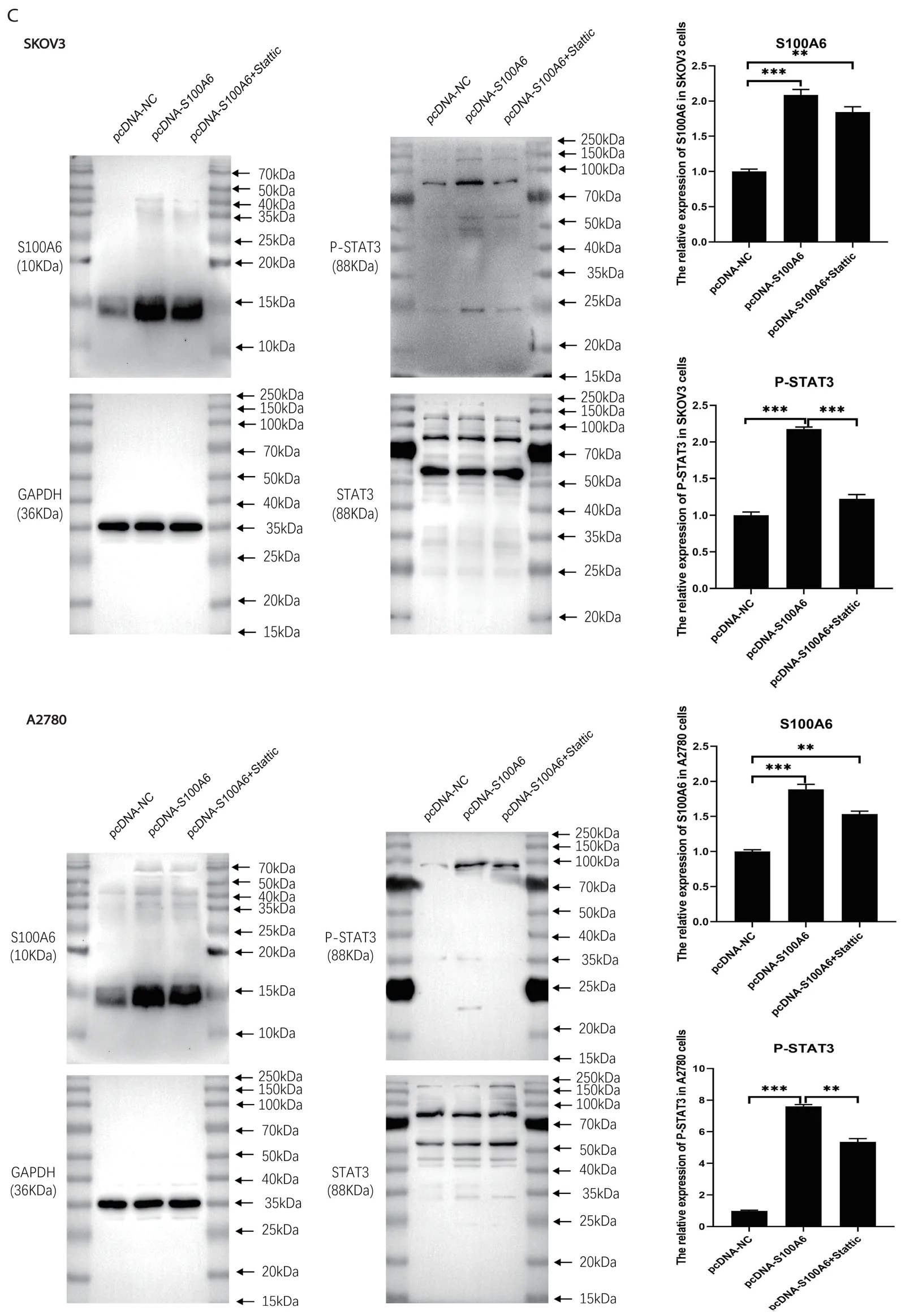
S100A6 activates the JAK-STAT pathway and Stattic reverses S100A6-promoted migration. (**A**) Western blot analysis showing that S100A6 knockdown significantly decreased phosphorylation levels of JAK2 and STAT3 in A2780 and SKOV3 cells, while total JAK2 and STAT3 protein levels remained unchanged. (**B**) Representative images and quantification of Transwell migration assays in SKOV3 and A2780 cells transfected with pcDNA-NC, pcDNA-S100A6, or pcDNA-S100A6 plus Stattic. Data are mean ± SD. *p* = 5.47 × 10^−6^ (SKOV3, pcDNA-S100A6 vs. pcDNA-NC), *p* = 9.75 × 10^−6^ (SKOV3, pcDNA-S100A6 + Stattic vs. pcDNA-S100A6); *p* = 1.75 × 10^−6^ (A2780, pcDNA-S100A6 vs. pcDNA-NC), *p* = 1.39 × 10^−5^ (A2780, pcDNA-S100A6 + Stattic vs. pcDNA-S100A6). (**C**) Western blot analysis and corresponding quantification of S100A6, p-STAT3, and total STAT3 protein levels in the three groups of both cell lines. GAPDH served as the loading control. * *p* < 0.05, ** *p* < 0.01, *** *p* < 0.001, **** *p* < 0.0001.

To elucidate the molecular mechanisms underlying the oncogenic effects of S100A6, we examined its impact on key signaling pathways involved in ovarian cancer progression. Western blot analysis was performed to assess pathway-related protein expression following S100A6 knockdown. The results demonstrated that silencing S100A6 significantly reduced the phosphorylation levels of JAK2 and STAT3, while total JAK2 and STAT3 protein levels remained unchanged (Figure 6A). To further validate the involvement of the JAK-STAT pathway in S100A6-mediated function, we overexpressed S100A6 in SKOV3 and A2780 cells and treated them with Stattic, a selective STAT3 inhibitor. Transwell migration assays showed that S100A6 overexpression increased cell migration, an effect effectively reversed by Stattic treatment (Figure 6B). Consistently, Western blot analysis revealed that S100A6 overexpression elevated p-STAT3 levels, while co-treatment with Stattic markedly reduced p-STAT3 without affecting total STAT3 expression (Figure 6C). These results suggest that S100A6 promotes ovarian cancer cell migration at least in part through STAT3 activation, and that pharmacological inhibition of STAT3 can abrogate this effect.

### 3.5. Let-7c-3p Directly Targets S100A6

**Figure 7:**
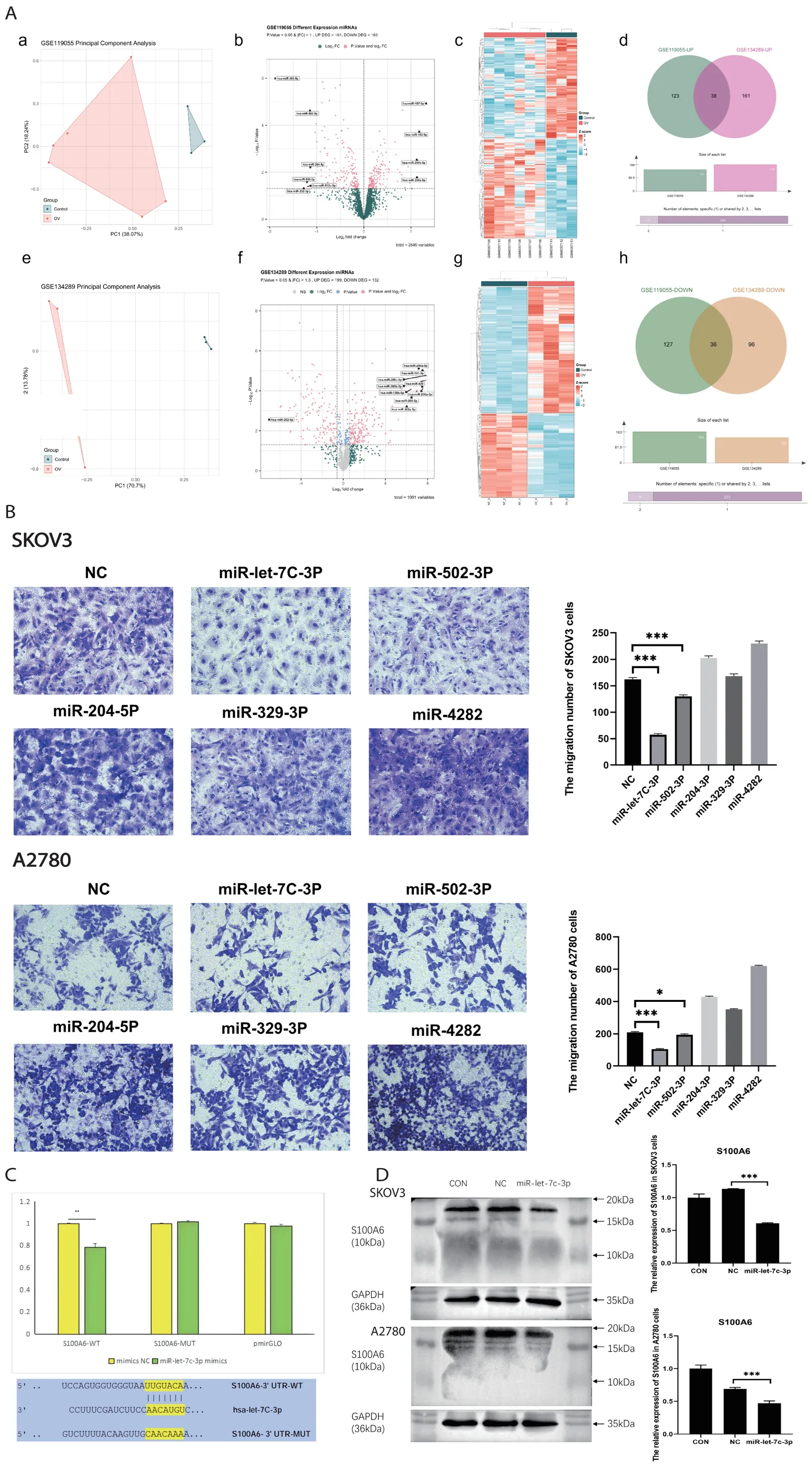
Identification of upstream miRNAs regulating S100A6 and validation of direct binding between miRNA Let-7c-3p and S100A6. (**A**) Differential expression analysis of ovarian cancer miRNA datasets GSE119055 and GSE134289. (**a**,**e**) PCA plots, (**b**,**f**) volcano plots, and (**c**,**g**) heatmaps illustrate differentially expressed miRNAs in the two datasets, and overlapping miRNAs were identified by cross-analysis (**d**,**h**) (|log_2_FC| ≥ 1, adjusted *p* < 0.05). (**B**) Migration assays showing the effects of candidate miRNA mimics on cell migration in SKOV3 and A2780 cells. Transfection with miR-Let-7c-3p and miR-502-3p significantly reduced migration compared with the NC group (*p* < 0.05). (**C**) Dual-luciferase reporter assays confirming that miR-Let-7c-3p directly binds to the 3′-UTR of S100A6. Mutation of the predicted binding site abolished the inhibitory effect of miR-Let-7c-3p on luciferase activity. (**D**) Western blot analysis demonstrates that miR-Let-7c-3p mimics significantly reduced S100A6 protein expression in A2780 and SKOV3 cells (*p* < 0.05). * *p* < 0.05, ** *p* < 0.01, *** *p* < 0.001.

Since microRNAs frequently regulate oncogenic signaling networks, we next explored potential miRNA regulators of S100A6. Bioinformatic analysis was performed to predict upstream miRNAs potentially targeting S100A6. Cell migration assays were subsequently used to screen candidate miRNAs, leading to the identification of miRNA Let-7c-3p as a focus of this study. Dual-luciferase reporter assays and Western blot analysis confirmed that Let-7c-3p directly binds to the 3′-untranslated region of S100A6 and negatively regulates its expression (Figure 7).

### 3.6. Let-7c-3p Suppresses Ovarian Cancer Progression Through the S100A6/JAK-STAT Axis

**Figure 8:**
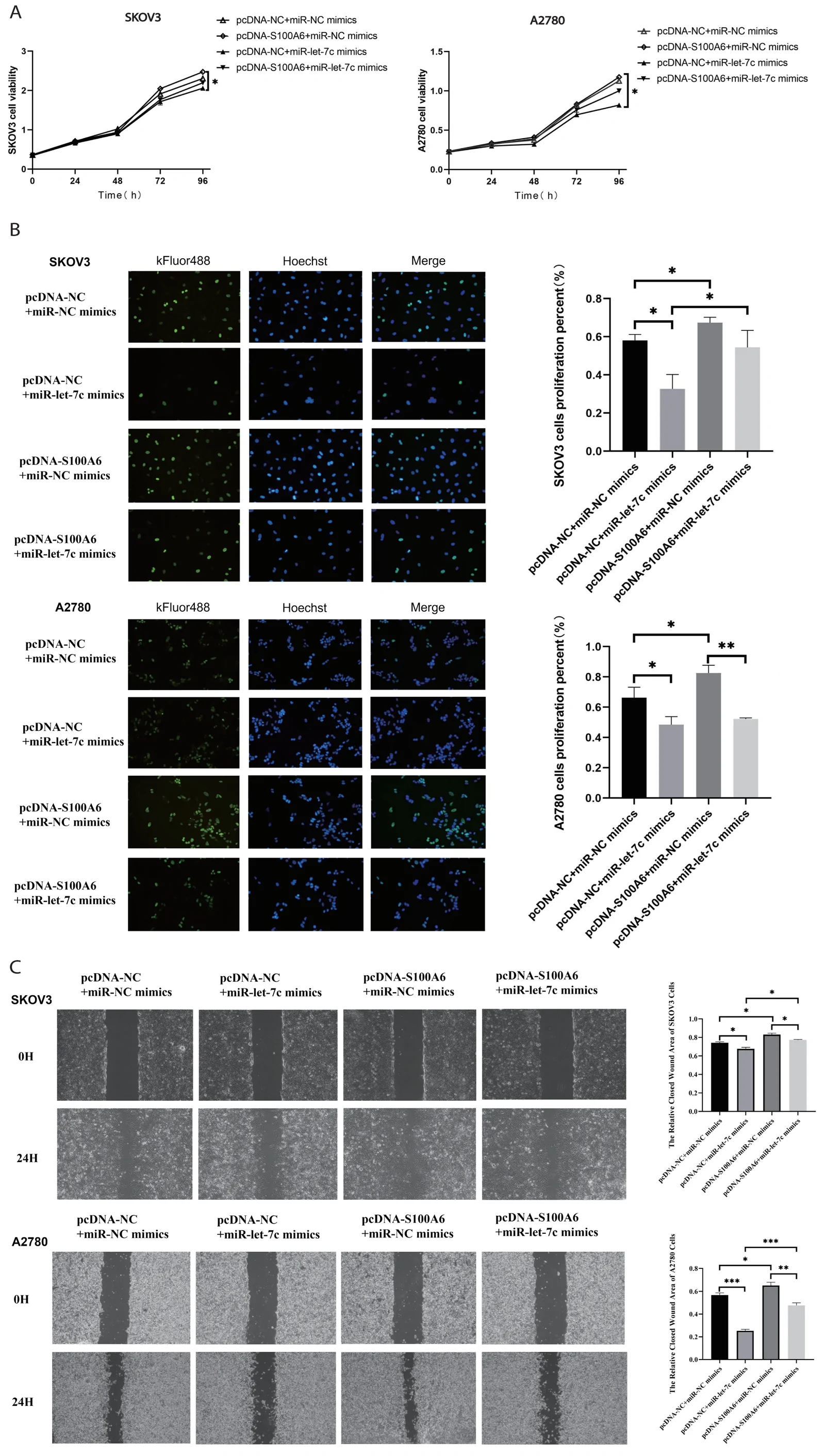

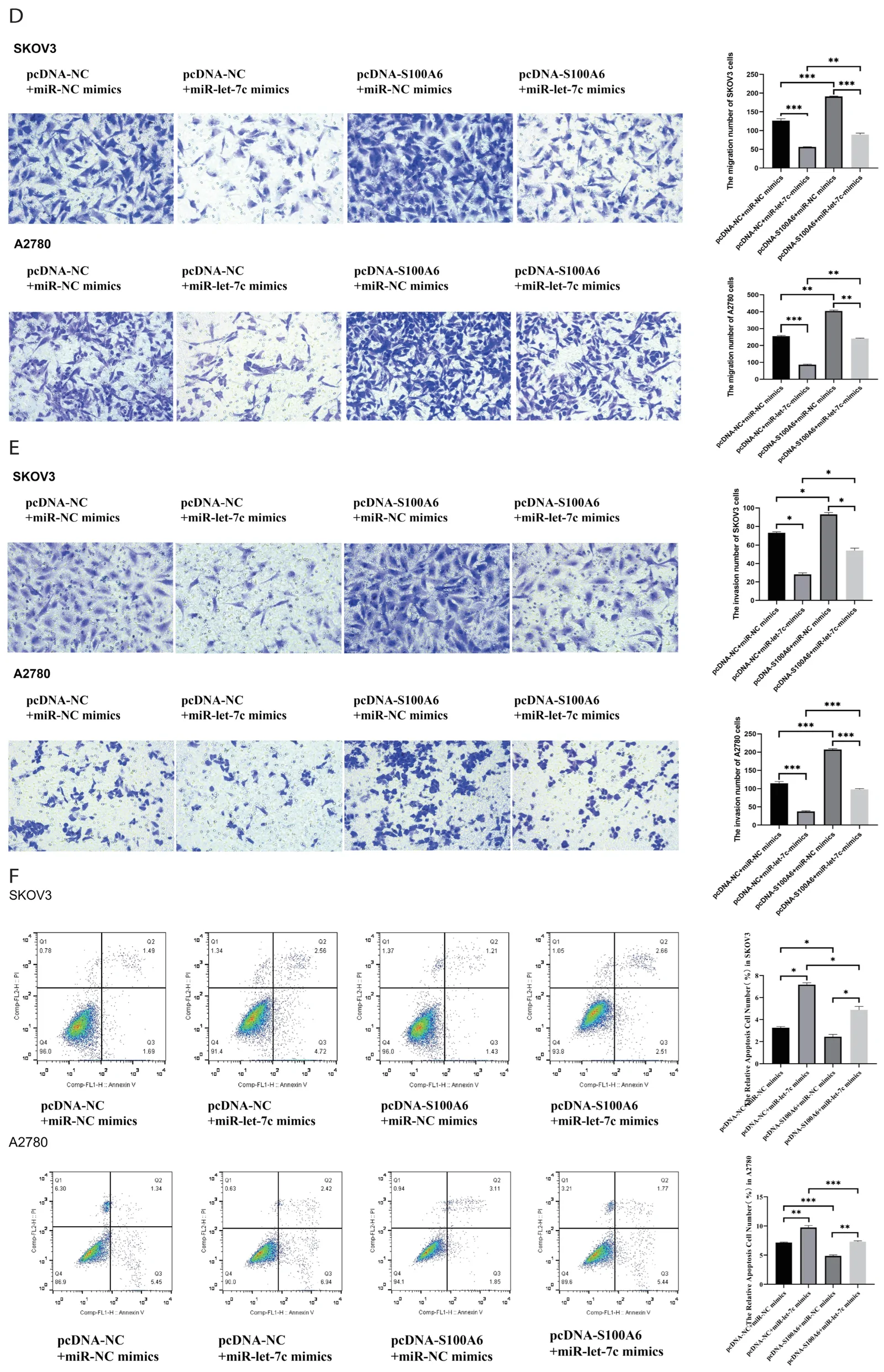
Rescue experiments validate that miRNA Let-7c-3p reverses S100A6-mediated oncogenic effects by downregulating S100A6. (**A**) CCK-8 assays showing the effects of Let-7c-3p and S100A6 on cell proliferation. (**B**) EdU assays evaluating proliferative capacity under different transfection conditions. (**C**) Wound-healing assays assessing migration ability. (**D**) Transwell migration assays confirming changes in migration. (**E**) Transwell invasion assays evaluating invasive capacity. (**F**) Flow cytometry analysis assessing apoptosis in ovarian cancer cells following rescue experiments. * *p* < 0.05, ** *p* < 0.01, *** *p* < 0.001.

To determine whether Let-7c-3p regulates ovarian cancer progression through S100A6, rescue experiments were performed in ovarian cancer cells using the following four transfection groups: pcDNA-NC + miR-NC mimics, pcDNA-S100A6 + miR-NC mimics, pcDNA-NC + miR-let-7c mimics, and pcDNA-S100A6 + miR-let-7c mimics. Functional assays including EdU staining, CCK-8, Transwell migration, wound healing, Transwell invasion, and flow cytometry apoptosis analyses were conducted in SKOV3 and A2780 cells. The results showed that transfection with pcDNA-S100A6 + miR-NC mimics significantly enhanced cell proliferation, migration, and invasion while reducing apoptosis compared to the pcDNA-NC + miR-NC mimics group (*p* < 0.05). Conversely, transfection with pcDNA-NC + miR-let-7c mimics significantly suppressed proliferation, migration, and invasion and promoted apoptosis compared to the pcDNA-NC + miR-NC mimics group (*p* < 0.05). Importantly, co-transfection with pcDNA-S100A6 + miR-let-7c mimics partially reversed the effects of S100A6 overexpression, demonstrating that Let-7c-3p attenuates S100A6-mediated oncogenic phenotypes (Figure 8).

### 3.7. miRNA Let-7c-3p Regulates S100A6 Expression and Modulates Cellular Functions Through the JAK-STAT Pathway

**Figure 9:**
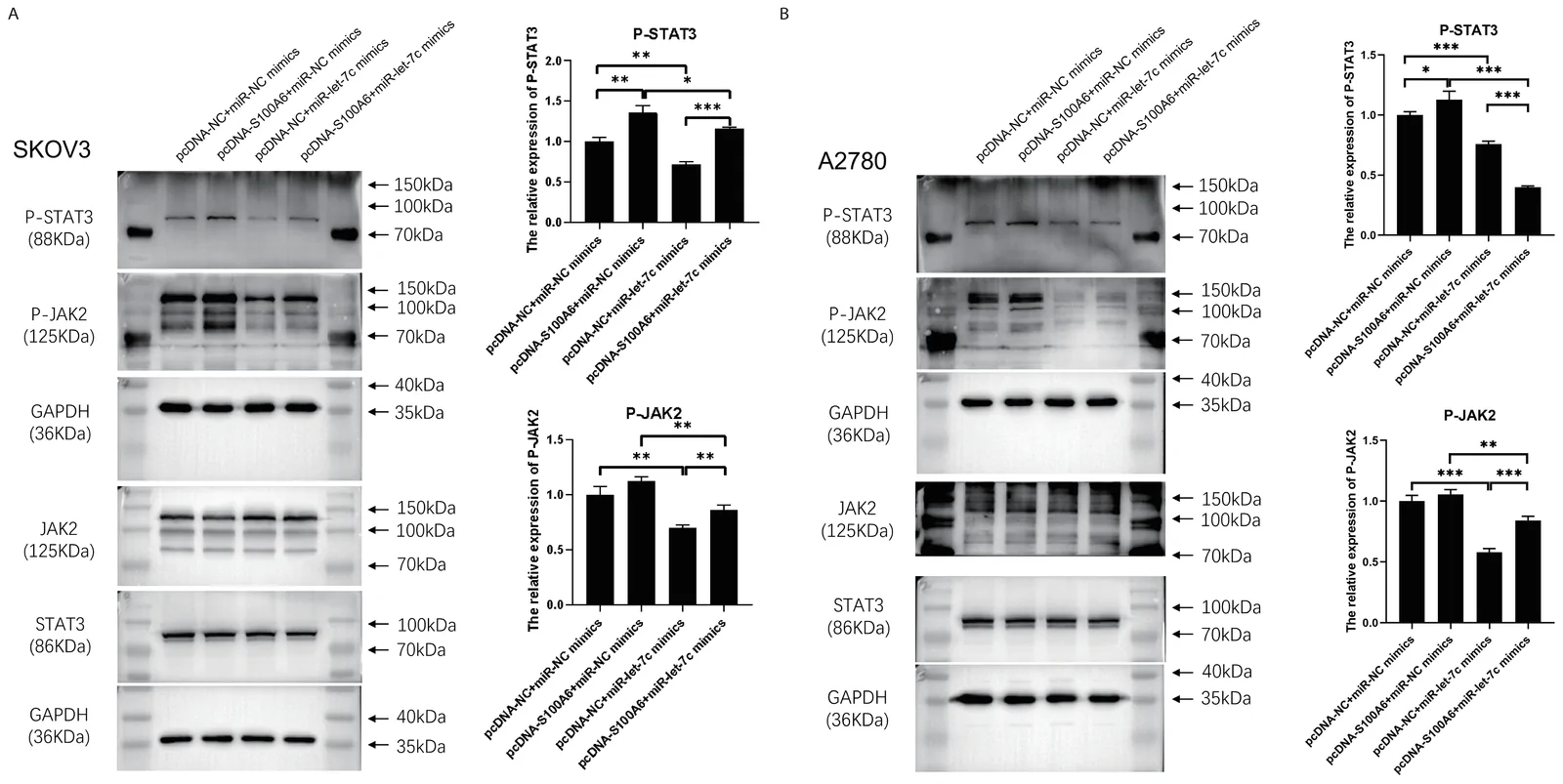
Western blot analysis confirms that miRNA Let-7c-3p regulates S100A6 expression and modulates cellular functions through the JAK-STAT pathway. Western blot analysis showing that S100A6 overexpression increased phosphorylation levels of JAK2 and STAT3, whereas Let-7c-3p mimics reduced pathway activation. Co-transfection partially reversed the S100A6-mediated activation of the JAK-STAT pathway. (**A**) and (**B**) Representative images of SKOV3 and A7280 cells, respectively. * *p* < 0.05, ** *p* < 0.01, *** *p* < 0.001.

To further confirm the involvement of the JAK-STAT pathway in Let-7c-3p-mediated regulation, Western blot analysis was performed on protein lysates from the rescue experiment groups. The results demonstrated that transfection with pcDNA-S100A6 + miR-NC mimics significantly increased the phosphorylation levels of JAK2 and STAT3 compared to the pcDNA-NC + miR-NC mimics group (*p* < 0.05). In contrast, transfection with pcDNA-NC + miR-let-7c mimics significantly decreased p-JAK2 and p-STAT3 levels compared to controls (*p* < 0.05). Notably, co-transfection with pcDNA-S100A6 + miR-let-7c mimics partially reversed the S100A6 overexpression-induced phosphorylation of JAK2 and STAT3 (Figure 9). These findings indicate that Let-7c-3p suppresses ovarian cancer cell malignancy at least in part through downregulation of S100A6 and subsequent inhibition of JAK-STAT signaling.

**Figure 10:**
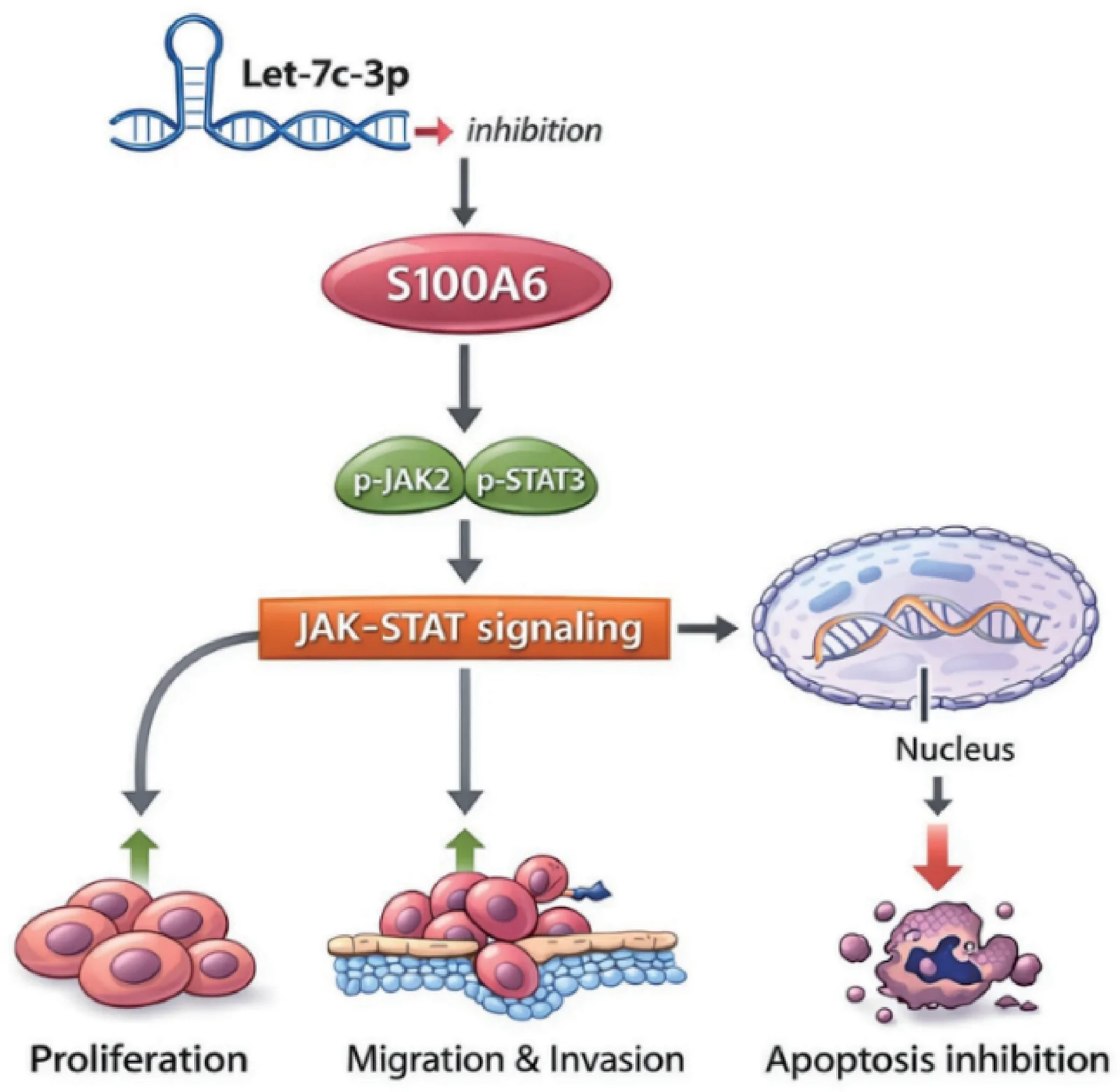
Proposed model of the Let-7c-3p/S100A6/JAK-STAT regulatory axis in ovarian cancer. Let-7c-3p directly targets the 3′-UTR of S100A6 and suppresses its expression. Reduced Let-7c-3p levels lead to increased S100A6 expression, which activates the JAK-STAT signaling pathway through enhanced phosphorylation of JAK2 and STAT3. Activation of this pathway promotes ovarian cancer cell proliferation, migration, and invasion while inhibiting apoptosis.

Based on these findings, we propose a regulatory model in which Let-7c-3p may suppress ovarian cancer progression, at least partially, through regulation of S100A6 expression and modulation of JAK-STAT pathway activity (Figure 10).

## 4. Discussion

Here, we show that Let-7c-3p directly represses S100A6, which in turn activates JAK–STAT signaling in ovarian cancer cells. Our data demonstrate that S100A6 is markedly upregulated in ovarian cancer cell lines and tumor tissues compared with normal ovarian epithelial cells. Functional assays showed that S100A6 promotes proliferation, migration, and invasion while inhibiting apoptosis in ovarian cancer cells. In vivo experiments further confirmed that S100A6 depletion suppresses tumor growth in xenograft models. Mechanistically, we found that S100A6 enhances the activation of the JAK-STAT signaling pathway, and that the tumor-suppressive microRNA Let-7c-3p directly targets S100A6 and attenuates its oncogenic effects. Consistent with these experimental findings, survival analysis using the Kaplan–Meier Plotter database revealed that elevated S100A6 expression is associated with shorter progression-free survival in ovarian cancer patients. Together, these results suggest that the Let-7c-3p/S100A6/JAK-STAT axis plays an important role in ovarian cancer progression.

Members of the S100 protein family have been increasingly recognized as regulators of tumor development and progression. Several S100 proteins, including S100A4 and S100A1, have been implicated in ovarian cancer metastasis and unfavorable clinical outcomes [8,9]. S100A6, a calcium-binding protein involved in cytoskeletal dynamics and cell cycle regulation, has been reported to be dysregulated in multiple malignancies [19,20,21]. However, its functional role and upstream regulatory mechanisms in ovarian cancer have remained incompletely understood. S100A6 appears to exert divergent functions in ovarian cancer—its elevated cytoplasmic and nuclear expression associates with better overall survival [12], yet it can also promote metastasis via HMGA2-USP30-mediated deubiquitination and stabilization [22]. Our findings extend previous observations by demonstrating that S100A6 not only contributes to malignant phenotypes of ovarian cancer cells but is also regulated by a specific microRNA-mediated mechanism. This provides additional evidence supporting the role of S100 family proteins as important mediators of tumor progression.

One of the key findings of this study is the identification of Let-7c-3p as a direct upstream regulator of S100A6. The Let-7 family is widely regarded as a tumor-suppressive miRNA family that regulates cell proliferation, differentiation, and oncogenic signaling [23,24,25,26]. Dysregulation of Let-7 members has been reported in various cancers, including ovarian cancer, where reduced Let-7 expression is associated with enhanced tumor aggressiveness and stemness [27]. In the present study, dual-luciferase reporter assays confirmed that Let-7c-3p directly binds to the 3′-UTR of S100A6, and gain-of-function experiments demonstrated that Let-7c-3p suppresses S100A6 expression and reverses its pro-tumorigenic effects. These findings suggest that loss of Let-7c-3p may contribute to S100A6 overexpression and subsequent activation of oncogenic signaling pathways in ovarian cancer.

Our results further indicate that S100A6 promotes malignant phenotypes partly through activation of the JAK-STAT signaling pathway. The JAK-STAT pathway is a well-established regulator of tumor cell proliferation, survival, and immune signaling, and persistent activation of STAT3 has been widely implicated in ovarian cancer progression and therapeutic resistance [28,29,30]. In this study, silencing S100A6 significantly reduced the phosphorylation levels of JAK2 and STAT3, whereas S100A6 overexpression enhanced pathway activation. Moreover, the inhibitory effects of Let-7c-3p on JAK-STAT signaling were partially reversed by S100A6 overexpression. This is further supported by the observation that pharmacological inhibition of STAT3 with Stattic reversed S100A6-promoted migration and reduced p-STAT3 levels, confirming that STAT3 phosphorylation is functionally required for S100A6-driven cell motility. These results suggest that S100A6 functions as a positive regulator of JAK-STAT signaling and that Let-7c-3p suppresses ovarian cancer progression, at least in part, through modulation of this pathway.

From a clinical perspective, the Let-7c-3p–S100A6–JAK–STAT regulatory connection identified in this study may have implications for ovarian cancer diagnosis and therapy. Aberrant activation of JAK-STAT signaling has been linked to poor prognosis and chemoresistance in ovarian cancer [31,32,33,34], and several JAK or STAT inhibitors are currently under clinical investigation [35,36]. Our data raise the possibility that tumors with elevated S100A6 expression may exhibit enhanced JAK-STAT activity and could potentially benefit from therapeutic strategies targeting this pathway. The prognostic link between S100A6 and shorter progression-free survival adds clinical weight to this notion. Although CA125 and HE4 remain the clinical mainstays, S100A6—and its upstream regulator Let-7c-3p—could offer complementary value for patient stratification. In addition, the ability of Let-7c-3p to reverse S100A6-mediated phenotypes also makes miRNA-based therapeutic approaches plausible, a strategy under active investigation in other cancer types. Translating these observations into clinical use will require prospective validation.

Nevertheless, several limitations should be acknowledged. First, although survival analysis using the Kaplan–Meier Plotter database indicated an association between S100A6 expression and progression-free survival, validation using independent transcriptomic cohorts was not performed. Moreover, the Kaplan–Meier Plotter analysis was based on an automatically selected best cutoff, which may introduce potential bias. Second, the precise molecular mechanism by which S100A6 activates the JAK-STAT pathway remains to be fully elucidated. It is possible that S100A6 regulates upstream cytokine signaling or interacts with other pathway components, which warrants further investigation. Third, given that S100A6 overexpression was only partially reversed by Let-7c-3p, other Let-7c-3p targets likely also contribute to its tumor-suppressive functions in ovarian cancer cells. Finally, additional clinical studies are required to determine whether the Let-7c-3p/S100A6 axis has predictive value for therapeutic response in ovarian cancer patients.

In conclusion, this study identifies a novel Let-7c-3p/S100A6/JAK-STAT regulatory axis that contributes to ovarian cancer progression. Our findings provide new insights into the molecular mechanisms underlying ovarian cancer development and suggest that targeting this pathway may represent a potential strategy for therapeutic intervention.

## 5. Conclusions

In summary, this study demonstrates that S100A6 is significantly upregulated in ovarian cancer and plays a critical oncogenic role by promoting cell proliferation, migration, invasion, and tumor growth while suppressing apoptosis. Mechanistically, S100A6 activates the JAK–STAT signaling pathway, at least in part, and its expression is directly repressed by the tumor-suppressive miRNA Let-7c-3p. Restoration of Let-7c-3p efficiently reverses S100A6-mediated malignant phenotypes and JAK-STAT activation. Together, these findings define a Let-7c-3p/S100A6/JAK-STAT connection in ovarian cancer, linking a tumor-suppressive miRNA to a pro-oncogenic calcium-binding protein and a downstream signaling cascade that drives malignant progression.

The identification of this connection not only deepens our understanding of ovarian cancer pathogenesis but also provides multiple potential targets for diagnostic and therapeutic intervention. Future studies should explore whether modulating Let-7c-3p or S100A6 levels can enhance chemosensitivity and whether this axis contributes to drug resistance in ovarian cancer patients. Targeting the Let-7c-3p/S100A6/JAK-STAT network may represent a promising strategy for the management of this devastating disease.

## Data Availability

The original contributions presented in this study are included in the article. Further correspondence should be addressed to the corresponding author. The raw data that support the findings of this article will be supplied by the authors upon request.
